# Comparison of a loading dose of dexmedetomidine combined with propofol or sevoflurane for hemodynamic changes during anesthesia maintenance: a prospective, randomized, double-blind, controlled clinical trial

**DOI:** 10.1186/s12871-018-0468-x

**Published:** 2018-01-24

**Authors:** Yuan Han, Liu Han, Mengmeng Dong, Qingchun Sun, Ke Ding, Zhenfeng Zhang, Junli Cao, Yueying Zhang

**Affiliations:** 1 Department of Anesthesiology, The Affiliated Hospital of Xuzhou Medical University, No.99 Huaihai West Road, Xuzhou, 221002 Jiangsu China; 2 Jiangsu Province Key Laboratory of Anesthesiology, Xuzhou Medical University, No. 209 Tongshan Road, Xuzhou, Jiangsu 221004 China

**Keywords:** Dexmedetomidine, Hemodynamic, Anesthesia maintenance, Propofol, Sevoflurane

## Abstract

**Background:**

There may be great individual variability in the hemodynamic effects of this dexmedetomidine. For this reason, the dose must be carefully adjusted to achieve the desired clinical effect. Whether a loading dose of dexmedetomidine produces hemodynamic side effects during the anesthesia maintenance is unknown. The aim of this study was to compare the effects of a loading dose of dexmedetomidine combined with propofol or sevoflurane on hemodynamics during anesthesia maintenance.

**Methods:**

Eighty-four patients who were scheduled for general surgery under balanced general anesthesia were randomly allocated into 4 groups (*n* = 21): the propofol and dexmedetomidine group, the sevoflurane and dexmedetomidine group, the propofol and normal saline group, or the sevoflurane and normal saline group. The hemodynamic indexes at the time of just before, 5 min after and the end of study drug infusion (dexmedetomidine or normal saline) were recorded. The incidence rates of increasing blood pressure at the end of study drug infusion (greater than 20% compared to baseline or before study drug infusion) were evaluated.

**Results:**

Mean arterial pressure increased significantly (*P* < 0.01) only in the propofol and dexmedetomidine group after intravenous dexmedetomidine compared administration. 80% of cases with propofol and dexmedetomidine had increased mean arterial blood pressure compared to only 5% of cases in the sevoflurane and dexmedetomidine group (*P* < 0.05). Heart rates in the propofol and dexmedetomidine and the sevoflurane and dexmedetomidine groups decreased significantly after dexmedetomidine infusion (*P* < 0.01).

**Conclusions:**

Intraoperative administration of a loading dose of dexmedetomidine combined with propofol in anesthesia maintenance proceeded a significant increase in blood pressure. In contrast, it combines with sevoflurane didn’t produce increased blood pressure. Meanwhile it is not unexpected that dexmedetomidine combined with propofol or sevofurance decreased heart rate, due to the known side effects of DEX. Therefore, dexmedetomidine should be used cautiously during the entire intravenous anesthesia maintenance period, especially during maintenance with propofol.

**Trial registration:**

Chinese Clinical Trial Registry, ChiCTR-IOR-17010423, registered on 13 January 2017.

## Background

Dexmedetomidine (DEX) is a highly selective α2 adrenoreceptor agonist that exhibits a unique sedative effect with minimal respiratory depression [1]. DEX also has many other advantages. For example, recent studies reported that a loading dose of dexmedetomidine during anesthesia maintenance promoted the analgesic effect of analgesic drugs, reduced postoperative restlessness and vomiting, and improved patients’ satisfaction with anesthesia [2–7]. Furthermore, clinical studies demonstrated that DEX also significantly decreased the incidence of delirium and the prevalence of complications in elderly patients admitted to the ICU [8, 9].

Dexmedetomidine exhibits a high ratio of specificity for the α2 receptor (α2/α1 1600:1), and it is a complete α2 agonist. However, DEX may active α1 adrenergic receptors on peripheral blood vessels and produce hemodynamic fluctuations [10]. Conflicting evidence-based medical research exists for the hemodynamic effects of DEX. Some studies argue that the use of dexmedetomidine prior to induction is associated with increased hypotension [11, 12], and other studies suggest that a large dose of dexmedetomidine at a high infusion speed is associated with hypertension [13], severe bradycardia [12], or cardiac arrest [14]. Therefore, DEX may produce side effects of elevated blood pressure, which may result in hypertension-related complications, especially in aged patients. Most studies observed the hemodynamic changes of DEX application prior to anesthesia, but few studies examined the hemodynamic effects of dexmedetomidine in combination with different general anesthetics during anesthesia maintenance.

In this study, we examined the effects of a loading dose of dexmedetomidine in combination with propofol or sevoflurane on hemodynamics during anesthesia maintenance.

## Methods

### Study protocol

This prospective, randomized, double-blind, controlled clinical trial was performed at The Affiliated Hospital of Xuzhou Medical University between July 2014 and March 2016. The Institutional Medical Ethics Committee of Xuzhou Medical University approved this study, which was performed in accordance with the approved guidelines. Written informed consents were obtained from all subjects. This trial is registered at the Chinese Clinical Trial Registry (ChiCTR-IOR-17010423). The sample size of the study was calculated based on previous studies [15, 16] and a pilot study. Twenty patients in each group were required to detect a difference between groups with a power of 0.8 and type I error of 0.05.

### Subjects

**Fig. 1:**
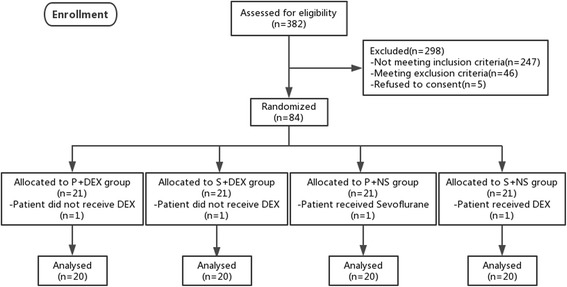
Enrollment

A total of 382 adults who were scheduled for general surgery under balanced general anesthesia with endotracheal intubation were asked to participate in this study. Eighty-four qualified patients were enrolled and randomly assigned to 4 groups: 2 test groups (propofol combined with DEX and sevoflurane combined with DEX) and 2 control groups (propofol combined with normal saline and sevoflurane combined with normal saline) (Fig. 1).

Patients who met the following criteria were included in this study: between 18 and 65 years old; American Society of Anesthesiologists (ASA) grade I or II; operation time expected to be greater than 1 h and less than 4 h; baseline blood pressure lower than 160/90 mmHg; heart rate greater than or equal to 60 beats/ min; no liver and renal dysfunction; and no abnormal anesthesia surgical history. We excluded patients whose ECGs revealed sinus tachycardia, sick sinus syndrome or atrioventricular block. Patients who were taking cardioactive or antihypertensive medications in the preoperative period were also excluded.

### Anesthesia

No patients received any preoperative drugs. Patients in all groups received an intravenous infusion of Compound Sodium Chloride Injection (10 ml/kg/h) on arrival, and systolic blood pressure (SBP), diastolic blood pressure (DBP), mean arterial pressure (MAP), heart rate (HR), bispectral index (BIS), electrocardiogram (ECG), oxygen saturation by pulse oximeter (SpO_2_) and end-tidal carbon dioxide (P_ET_CO_2_) were monitored continuously.

Patients in all groups were given midazolam (0.05 mg/kg), etomidate (0.3 mg/kg), fentanyl (3 μg /kg), cisatracurium (0.2 mg/kg) and remifentanil (1 μg/kg) at induction. Patients were ventilated with oxygen flow 1–2.0 L/min, tidal volume of 8–10 ml/kg immediately after intubation. Anesthesia was subsequently maintained using the different methods of the two test groups and two control groups and adjusted to maintain an acceptable blood pressure and BIS value within 40–60. Cisatracurium (0.05 mg/kg) was used intermittently for muscle relaxation, and fluids were given based on calculations of intraoperative fluid volume.

#### Two test groups

Propofol and DEX group (P+ DEX group): Intravenous infusion was switched to a maintenance syringe pump at a rate of 4–6 mg/kg/h for propofol and 0.3 μg/kg/min for remifentanil.

Sevoflurane and DEX group (S+ DEX group): Anesthesia maintenance was provided using sevoflurane 1–2% and remifentanil 0.3 μg/kg/min.

A loading dose of 1 μg/kg dexmedetomidine hydrochloride injection (Ai Beining, Jiangsu Hengrui Medicine Co., Ltd., 200 μg added to normal saline and adjusted to a concentration of 4 μg/ml) was intravenously infused over 10 min approximately 30 min before the end of surgery in the two test groups. The doses of anesthesia maintenance medication were not altered during DEX infusion. DEX infusion was immediately stopped if a patient’s heart rate was less than 40 beats/min or blood pressure was higher than 180/100 mmHg, in which case atropine (0.5 mg) or urapidil (15 mg) was intravenously injected.

#### Two control groups

Propofol and Normal Saline group (P + NS group): Anesthesia maintenance method was the same as the P+ DEX group: propofol (4–5 mg/kg/h) and remifentanil (0.3 μg/kg/min).

Sevoflurane and Normal Saline group (S + NS group): Sevoflurane 2–3% and remifentanil (0.3 μg/kg/min) were provided during anesthesia maintenance in the same manner as the S+ DEX group.

**Fig. 2:**
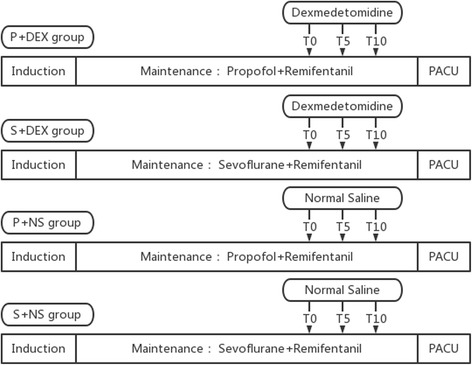
Study methods for each group

Patients in the above two control groups received volume-matched normal saline as a continuous intravenous infusion over 10 min approximately 30 min before the end of surgery (Fig. 2).

### Measurements

Patients’ demographic information was collected on admission. Intraoperative blood pressure, heart rate, drug use, and fluid administration were recorded in the electronic anesthesia record. Hemodynamic indexes, including SBP, DBP, MAP and HR, were recorded in all groups just before (T0), 5 min after (T5) and the end of (T10) continuous intravenous infusions of the study drug (DEX or normal saline). The incidence rates of increasing blood pressure at T10 (greater than 20% of the baseline or T0) were evaluated in both test groups.

### Randomization and blinding

All patients were assigned using a computer-generated random number table into 1 of 4 groups (*n* = 21): P+ DEX group, S+ DEX group, P + NS group and S + NS group. Group allocation was concealed until just before anesthesia, when investigators opened sequentially numbered opaque envelopes. The anesthesiologist was aware of the randomization but did not participate in any other part of the study. A blinded specialized investigator, who was also responsible for data analysis, gathered the intraoperative data. Patients were blinded to their allocation during the entire trial. Surgeons were also blinded to randomization.

### Statistical analysis

Data analyses first entailed characterization of participants using descriptive and summary statistics (means (SD) for SBP, DBP, MAP and HR; n (%) for incidence rate of increasing blood pressure). Data of the 4 groups were compared using one-way ANOVA (for basic demographic data and surgery/anesthesia-related information, Table 1), two-way ANOVA (for hemodynamic changes during intravenous infusion of study drug, Table 2), and Chi-square test. ANOVA and post hoc tests were used to compare data within a group, and Chi-square and Fisher exact test analyses were used to compare proportions. Sample size calculations were based on a power of 80% with 5% α-error and a β-error of 0.2. All *P* values given are based on 2-tailed tests, and a *P* value less than 0.05 was considered statistically significant. Statistical analyses of data were generated using Statistical Package for Social Science, SPSS, version 16.0 (IBM, New York, NY).

**Table 1:** Basic demographic data and surgery/anesthesia-related information

	P + DEX group (*n* = 20)	S + DEX group (*n* = 20)	P + NS group (*n* = 20)	S + NS group (*n* = 20)	*P* value
Age (y)	47(15)	42(11)	45(15)	49(15)	0.488
Gender, F/M (n)	13/7	9/11	11/9	9/11	0.531
Weight (kg)	66(15)	68(12)	67(9)	67(7)	0.924
BMI (kg/m^2^)	24(5)	24(3)	25(3)	24(3)	0.986
ASA, I/II (n)	5/15	5/15	5/15	4/16	0.976
Baseline SBP (mmHg)	130(18)	131(15)	128(12)	133(12)	0.807
Baseline DBP (mmHg)	72(10)	77(9)	73(7)	75(11)	0.251
Baseline MBP (mmHg)	91(11)	95(10)	91(8)	94(10)	0.453
Baseline HR (bpm)	78(6)	77(6)	74(8)	73(8)	0.133
BIS	48(5)	47(5)	45(5)	47(6)	0.275
Anesthesia time (min)*^#^	116(38)	146(37)	112(35)	149(41)	0.002
Operation time (min)*^#^	105(37)	131(30)	100(36)	129(38)	0.018
Type of surgery (n[%])					
Thyroid	12(60%)	10(50%)	9(45%)	7(35%)	0.456
Breast	8(40%)	10(50%)	11(55%)	13(65%)	

Values are means (SD) or number. *P < 0.05, P + DEX group vs. S + DEX group; ^#^*P* < 0.05, P + NS group vs. S + NS group;

*F* Female, *M* Male, *BMI* Body mass index, *ASA* American Society of Anesthesiologists, *SBP* Systolic blood pressure, *DBP* Diastolic blood pressure, *MAP* Mean arterial pressure; *HR* Heart rate, *T0* Just before continuous intravenous infusion of study drug

**Table 2:** Hemodynamic values during intravenous infusion of study drug (DEX or NS)

	P + DEX group (*n* = 20)	S + DEX group (*n* = 20)	P + NS group (*n* = 20)	S + NS group (*n* = 20)
SBP (mmHg)				
T0	108(16)	119(13)	120(16)	125(11)
T5	140(20)^^	122(13)	120(17)	124(12)
T10	153(23)^^	126(17)	122(15)	124(10)
ΔT5	32(17)**^##^	3(10)	0(4)	-1(5)
ΔT10	45(20)**^##^	6(16)	2(6)	-1(5)
DBP (mmHg)				
T0	63(10)	70(10)	65(12)	70(7)
T5	79(11)^^	71(7)	65(13)	70(8)
T10	88(11)^^	71(11)	66(9)	70(7)
ΔT5	17(10)**^##^	2(9)	0(2)	0(3)
ΔT10	25(11)**^##^	2(14)	1(5)	0(4)
MAP (mmHg)				
T0	78(11)	86(9)	83(13)	88(7)
T5	100(13)^^	88(7)	83(14)	88(9)
T10	109(13)^^	90(11)	84(10)	88(7)
ΔT5	22(11)**^##^	2(9)	0(2)	0(3)
ΔT10	32(13)**^##^	4(14)	1(5)	0(4)
HR (bpm)				
T0	64(11)	72(9)	67(10)	64(7)
T5	55(5)^^	59(8)^^	67(11)	64(6)
T10	53(4)^^	58(9)^^	66(11)	63(7)
ΔT5	−10(9)^##^	−13(10)^&&^	0(3)	−1(3)
ΔT10	−11(11)^##^	−14(10)^&&^	0(4)	−1(3)

Values are means (SDs). ^^*P* < 0.01, compared to T0; **P < 0.01, P + DEX group vs. S + DEX group; ^##^*P* < 0.01, P+ DEX group vs. P + NS group; ^&&^*P* < 0.01, S+ DEX group vs. S + NS group

*SBP* Systolic blood pressure, *DBP* Diastolic blood pressure, *MAP* Mean arterial pressure, *HR* Heart rate, *T0* Just before continuous intravenous infusion of study drug, *T5* 5 min after continuous intravenous infusion of study drug, *T10* The end of continuous intravenous infusion of study drug, ΔT5 = T5-T0, ΔT10 = T10-T0

## Results

### Enrollment

A total of 382 patients were screened for study participation between July 2014 and March 2016. A total of 247 patients did not meet the inclusion criteria, 46 patients satisfied the exclusion criteria, and 5 patients refused to consent. These patients were excluded from the study. Thus, eighty-four patients were enrolled into the study and randomly assigned to the 4 groups. One patient assigned to the P+ DEX group and one patient assigned to the S+ DEX group were withdrawn without receiving DEX. One patient assigned to the P + NS group was excluded because they received sevoflurane, and one patient assigned to the S + NS group was excluded because they received DEX. These four patients were excluded from the study. Therefore, the records of 80 patients in four groups were available for the final analysis (Fig. 1).

### Demographic data

The demographic characteristics, including age, sex, weight, BMI, ASA class, baseline SBP, DBP, MAP and HR, were comparable between the 4 randomization groups, except that the anesthesia time and operation time in the S+ DEX and S + NS groups were longer than the P+ DEX and P + NS groups (Table 1). None of the included patients were taking cardioactive or antihypertensive medication in the preoperative period.

### Outcomes

#### Change in blood pressure

SBP, DBP and MAP increased significantly in the P+ DEX group after continuous intravenous infusion of a loading dose of DEX at T5 compared to T0 (*P* < 0.01). Furthermore, SBP, DBP and MAP values were much higher in the P+ DEX group at T10 than at T0. However, SBP, DBP and MAP were not increased at T5 and T10 in the S+ DEX, P+ NS or S + NS groups compared with T0 (Table 2).

**Fig. 3:**
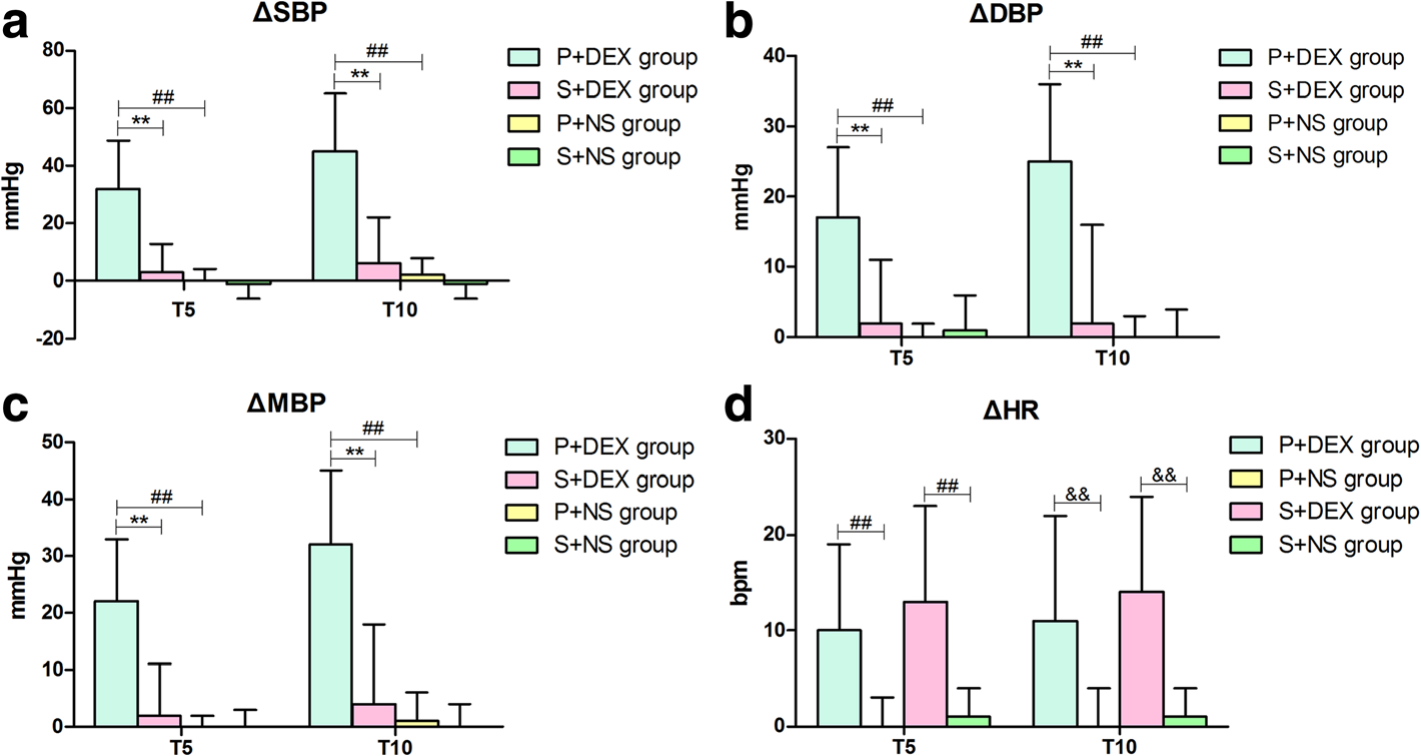
SBP, DBP, MAP and HR changes. **a**, ΔSBP changes of test and control groups. **b**, ΔDBP changes of test and control groups. **c**, ΔMBP changes of test and control groups. **d**, ΔHR changes of test and control groups. Δ = (T5 or T10)-T0, T0 = just before continuous intravenous infusion of study drug, T5 = 5 min after continuous intravenous infusion of study drug, T10 = the end of continuous intravenous infusion of study drug.**P < 0.01, P + DEX group vs. S + DEX group; ^##^*P* < 0.01, P+ DEX group vs. P + NS group; ^&&^*P* < 0.01, S+ DEX group vs. S + NS group. SBP=Systolic blood pressure, DBP = Diastolic blood pressure, MAP = Mean arterial pressure, HR = Heart rate

ΔSBP, ΔDBP and ΔMAP were larger in the P + DEX group at T5 and T10 compared to the S + DEX group (*P* < 0.01). ΔSBP, ΔDBP and ΔMAP were also larger in the P + DEX group compared to the P + NS group (*P* < 0.01). However, there were no differences between the S+ DEX, P + NS and S + NS groups in ΔSBP, ΔDBP and ΔMAP (Table 2, Fig. 3a–c).

**Fig. 4:**
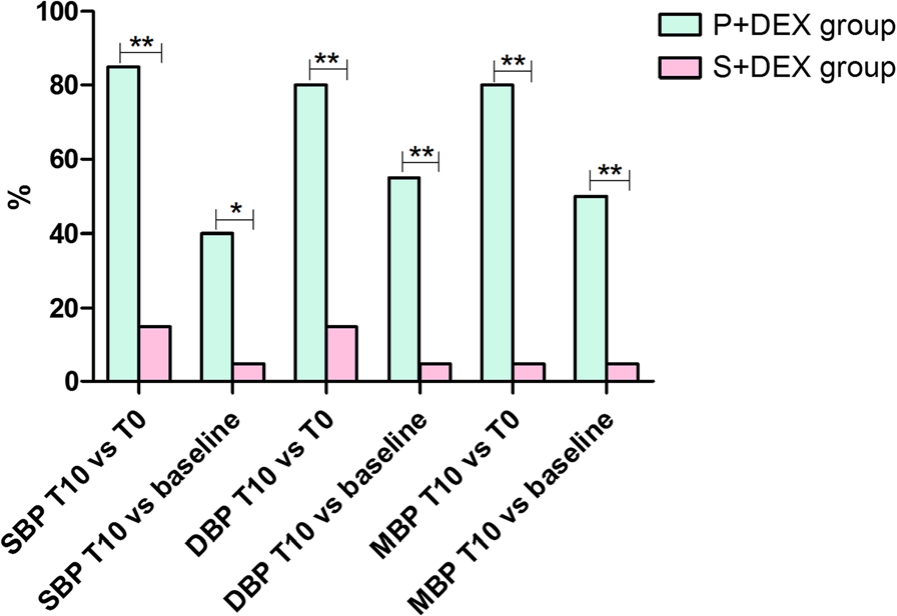
Incidence rates of increasing blood pressure in test groups. SBP=Systolic blood pressure, DBP = Diastolic blood pressure, MAP = Mean arterial pressure, T0 = just before continuous intravenous infusion of study drug, T10 = the end of continuous intravenous infusion of study drug

We also analyzed the incidence of increased blood pressure (increased greater than 20% compared to baseline or T0 values) in the test groups. The incidence rates of SBP, DBP and MAP in the P+ DEX group were much higher than those in S+ DEX group (*P* < 0.05). The respective differences in SBP, DBP and MAP between both test groups were significant using the chi-square test (Table 3, Fig. 4).

**Table 3:** Incidence rates of increasing blood pressure in test groups

	P + DEX group (*n* = 20)	S + DEX group (*n* = 20)	Chi-square test *P* value
SBP increased significantly at T10			
compared to T0	17(85%)	3(15%)	<0.001
compared to baseline	8(40%)	1(5%)	0.023
DBP increased significantly at T10			
compared to T0	16(80%)	3(15%)	<0.001
compared to baseline	11(55%)	1(5%)	0.001
MAP increased significantly at T10			
compared to T0	16(80%)	1(5%)	<0.001
compared to baseline	10(50%)	1(5%)	0.001

Values are numbers (proportion)

*SBP* Systolic blood pressure, *DBP* Diastolic blood pressure, *MAP* Mean arterial pressure, *T0* Just before continuous intravenous infusion of study drug, *T10* The end of continuous intravenous infusion of study drug

These outcomes demonstrated that the administration of a loading dose of DEX combined with propofol during anesthetic maintenance may lead to a significant increase in blood pressure compared with DEX combined with sevoflurane.

#### Change in heart rate

Heart rates in the P+ DEX and S+ DEX groups decreased significantly after infusion of a loading dose of DEX at T5 and T10 compared to at T0 (*P* < 0.01), and heart rates at T10 was much lower than those at T5. There were no differences between time points in the P + NS group and S + NS group (Table 2). Heart rates decreased significantly in the P + DEX group and S+ DEX group compared to the control P + NS and S+ NS groups (*P* < 0.01, Table 2, Fig. 3d).

These outcomes indicated that the administration of a loading dose of DEX combined with propofol or sevoflurane in anesthesia maintenance decreased heart rate.

No other adverse events related to the study interventions were observed.

## Discussion

It is important for anesthesiologists to maintain the perioperative hemodynamics of patients, especially stability of arterial blood pressure. The effects of anesthetics on hemodynamics should also be monitored. The recommend usage of DEX is commonly initiated with a loading dose before the induction of anesthesia and followed by a maintenance infusion during the maintenance of anesthesia, and it usually exhibits a central anti-sympathetic effect that may deepen the depth of anesthesia, spare the dose of anesthetics and reduce medical costs. Recently, more and more studies focus on evaluating the effects of loading dexmedetomidine during anesthesia maintenance and found that if it was initiated just half hours before the end of surgery, DEX could promote the analgesic effect of analgesic drugs, reduced postoperative restlessness and vomiting, and improved patients’ satisfaction with anesthesia [2–7]. Furthermore, other clinical studies demonstrated that DEX initiated just half hours before the end of surgery also significantly decreased the incidence of delirium and the prevalence of complications in elderly patients admitted to the ICU [8, 9]. However, there are many conflicting evidence-based medical evidences exist for the hemodynamic effects of DEX. Besides the good aspect of DEX, it may induce hemodynamic fluctuations, even it is a highly selective α2-adrenergic agonist [10]. Therefore, it is why we focus on the evaluation of its safety, especially for giving a loading DEX during the maintenance of anesthesia.

The present study found that DEX combined with propofol during anesthesia maintenance period increased systolic and diastolic blood pressure 5 min after DEX administration compared to propofol control group and significantly increased blood pressure 10 min after DEX infusion. The incidence rate of MAP increases at the end of DEX infusion were significantly higher in the propofol test group than the normal saline control group compared to T0 (16/20, 80%) and baseline (10/20, 50%). These data indicate that the loading dose of DEX during the propofol infusion produced a serious increase in blood pressure. However, the increase in blood pressure induced by DEX infusion was not statistically significant in the sevoflurane test group compared with the sevoflurane control group.

We also investigated whether the different anesthesia maintenance methods would affect the DEX infusion-induced changes in blood pressure. We compared blood pressure changes between the P + DEX and S + DEX groups. Notably, we found that ΔMAP in the P + DEX group was much higher than the S + DEX group, and the incidence rates of blood pressure increase after DEX infusion were also significantly higher in the P + DEX group than the S + DEX group. These data suggest that anesthesia maintenance medicines may be used carefully and chosen appropriately in patients with high risk of cardiovascular and cerebrovascular accidents, such as aneurysm and myocardial ischemia.

We further focused on the mechanisms of DEX-induced blood pressure increases and blood pressure stability in the sevoflurane group. Firstly, the reason might be that propofol and sevoflurane show different roles in inhibition of aortic baroreceptor reflex. Studies showed that propofol may reset or may inhibit the baroreflex, reducing the tachycardic response to hypotension, for heart rate does not change significantly after hypotension [17, 18]. But when seveflurane induces the hypotension, usually the heart rate will go up. Thus, when faced the hypertension, baroreflex will be activated and maintain hemodynamic stability in seveflurane group; but not for the propofol group.

Another speculation is that sevoflurane could produce stronger vasodilated role and potentially attenuate DEX induced vasoconstriction. Previous studies found that postsynaptic α2 adrenoreceptors on peripheral blood vessels produced vasoconstriction, and the increase in blood pressure was likely due to the vasoconstrictive effects of DEX stimulation of peripheral α2 receptors [15, 19]; however, sevoflurane could produce vasodilation and potentially attenuate this vasoconstriction [20]. This mechanism may explain the relatively stable blood pressure in the sevoflurane group compared to the propofol group. However, we do not know the cause DEX-induced blood pressure elevation in combination with propofol because the literature reports that propofol decreased blood pressure primarily via a decrease in systemic vascular resistance [21].

Also, there are many other intervention factors can induce the difference between the groups. Firstly, blood volume status may play an important role in the maintenance of blood pressure, however this data was not collected within our protocol. There may be an unknown interaction between DEX and the anesthetics, therefore we recommended further evidenced-based medicine and laboratory studies in this area.

Secondly, we found that the propofol and DEX group had a lower blood pressure value than the sevoflurane groups at T0, it may lay the opportunity to be an intervention of hemodynamic between the groups. Actually, we have considered that if we only compare average value of hemodynamic between groups at T1 or T2, the preexisting hemodynamic difference between groups would be an important intervention factor. So, we measured and analyzed the hemodynamic difference between T0 and T1/T2 of each individual, which can minimize the preexisting hemodynamic different between groups to a great extent.

Thirdly, we also noticed that both the operation and anesthesia time were significantly longer in sevoflurane group than propofol group. For our trial is a double-blind, randomized, controlled clinical trial, the length of surgery was already determined and actually uncontrollable. However, the possibility that the length of surgery or anesthesia is a cause of hemodynamic differences between groups is less likely. During the surgery, the most important reasons for interfering hemodynamic stability is the new loading drugs, blood volume status and some special surgery procedures. The length of surgery may only have some indirectly interference with the blood pressure. In our trial, the most likely reason for the hemodynamic changes we observed is most likely to be the DEX. In one hand, the hemodynamic changes we observed in the trials is just link with the loading of DEX, but not in the saline groups. Even the operation and anesthesia time were significantly different in the saline group, there are no the blood pressure between these groups. Meanwhile, the blood pressure is increased significantly in DEX and proforol group. In the other hand, DEX related hemodynamic changes in each group were compared with its own base value, and those observed changes just happened after DEX infusion, but didn’t link with the length of the surgery. Therefore, although there are significant differences operation/anesthesia time between the groups, this hemodynamic change is most likely associated with DEX.

In addition, the time point that we conducted the trials as 30 min before the end of the surgery might be a better choice, and also have the post-operative benefits for patients. Otherwise, for example, if it is conducted at the very beginning, many kinds of induce anesthesia drugs may open the possibility of much more other factors contributing to the blood pressure changes than at this time point. If it is conducted at the beginning or during the main surgery procedure, the special surgery procedures themselves would interfere the blood pressure.

The results of this study demonstrated that a loading dose of DEX during anesthesia maintenance decreased heart rate regardless of the maintenance methods of anesthesia, primarily via stimulation of the vagus nerves. Therefore, the use of DEX during anesthesia carries the risk of a decrease in heart rate, but the degree of decrease did not lead to severe hemodynamic disorders.

There are several other limitations to this study. First, the sample size was relatively small, and this study was a single-center clinical trial. Second, the study was also limited by our inability to blind the anesthesiologists to the anesthesia maintenance method randomization because the administration of propofol or sevoflurane is obvious, which may introduce potential bias to the intraoperative anesthesia management. Third, there was no comparison of postoperative long-term hemodynamic indexes.

## Conclusions

In summary, intraoperative administration of a loading dose of DEX combined with propofol or sevoflurane during anesthesia maintenance produced a decrease in heart rate, but DEX in combination with propofol produced a significant increase in blood pressure that clinicians should closely monitor. Therefore, DEX should be used cautiously during intravenous anesthesia maintenance period, especially in patients with primary hypertension, to avoid serious hemodynamic changes.

## Abbreviations

ASA: American Society of Anesthesiologists
BIS: Bispectral index
BMI: Body mass index
DBP: Diastolic blood pressure
DEX: Dexmedetomidine
ECG: Electrocardiogram
F: Female
HR: Heart rate
M: Male
MAP: Mean arterial pressure
P: Propofol
PETCO2: End-tidal carbon dioxide
S: Sevoflurane
SBP: Systolic blood pressure
SpO_2_: Oxygen saturation by pulse oximeter
T0: Just before continuous intravenous infusion of study drug
T10: The end of continuous intravenous infusion of study drug
T5: 5 min after continuous intravenous infusion of study drug
ΔT10: T10-T0
ΔT5: T5-T0
